# Bis(guanidinium) chloranilate

**DOI:** 10.1107/S1600536811036373

**Published:** 2011-09-14

**Authors:** Konstantin A. Udachin, Md. Badruz Zaman, John A. Ripmeester

**Affiliations:** a Steacie Institute for Molecular Sciences, National Research Council of Canada, 100 Sussex, Ottawa, Ontario, K1A 0R6, Canada; b Center of Excellence for Research in Engineering Materials, Faculty of Engineering, King Saud University, Riyadh 11421, Saudi Arabia

## Abstract

The asymmetric unit of the title co-crystal, 2CH_6_N_3_^+^·C_6_Cl_2_O_4_^2−^, contains one half of a chloranilate anion and one guanidinium cation, which are connected by strong N—H⋯O hydrogen bonds into a two-dimensional network.

## Related literature

For organic co-crystals containing 2,5-dihy­droxy-3,6-dichloro-1,4-benzoquinone (chloranilic acid), see: Andersen & Andersen (1975 ▶); Horiuchi *et al.* (2005 ▶, 2007 ▶); Zaman *et al.* (1999*a* ▶,*b* ▶, 2010 ▶). For inorganic co-ordination polymers containing chloranilic acid, see: Kitagawa *et al.* (2002 ▶). For guanidine and guanidinium structures, see: Abrahams *et al.* (2004 ▶, 2005 ▶); Best *et al.* (2003 ▶); Said *et al.* (2006 ▶); Smith & Wermuth (2010 ▶, 2011 ▶). 
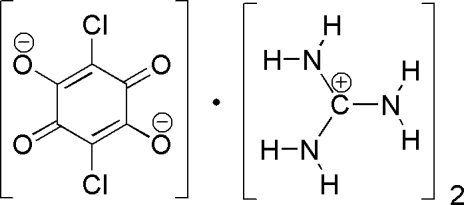


## Experimental

### 

#### Crystal data

2CH_6_N_3_^+^·C_6_Cl_2_O_4_^2−^
*M* *_r_* = 327.14
Monoclinic, 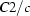

*a* = 19.5224 (14) Å
*b* = 3.7316 (3) Å
*c* = 18.4103 (14) Å
β = 116.087 (1)°
*V* = 1204.56 (16) Å^3^
*Z* = 4
Mo *K*α radiation
μ = 0.57 mm^−1^
*T* = 173 K
0.45 × 0.40 × 0.30 mm

#### Data collection

Bruker SMART 1000 CCD diffractometer
Absorption correction: multi-scan (*SADABS*, Sheldrick, 1996 ▶) *T*_min_ = 0.785, *T*_max_ = 0.849
6965 measured reflections
1674 independent reflections
1525 reflections with *I* > 2σ(*I*)
*R*_int_ = 0.020

#### Refinement

*R*[*F* ^2^ > 2σ(*F* ^2^)] = 0.026
*wR*(*F* ^2^) = 0.078
*S* = 1.08
1674 reflections
116 parameters
All H-atom parameters refined
Δρ_max_ = 0.47 e Å^−3^
Δρ_min_ = −0.25 e Å^−3^

### 

Data collection: *SMART* (Bruker, 2003 ▶); cell refinement: *SAINT-Plus* (Bruker, 2003 ▶); data reduction: *SAINT-Plus*; program(s) used to solve structure: *SHELXS97* (Sheldrick, 2008 ▶); program(s) used to refine structure: *SHELXL97* (Sheldrick, 2008 ▶); molecular graphics: *ATOMS* (Dowty, 1999 ▶); software used to prepare material for publication: *SHELXL97*.

## Supplementary Material

Crystal structure: contains datablock(s) I, global. DOI: 10.1107/S1600536811036373/vm2118sup1.cif

Structure factors: contains datablock(s) I. DOI: 10.1107/S1600536811036373/vm2118Isup2.hkl

Supplementary material file. DOI: 10.1107/S1600536811036373/vm2118Isup3.cml

Additional supplementary materials:  crystallographic information; 3D view; checkCIF report

## Figures and Tables

**Table 1:** Hydrogen-bond geometry (Å, °)

*D*—H⋯*A*	*D*—H	H⋯*A*	*D*⋯*A*	*D*—H⋯*A*
N1—H2⋯O3	0.85 (2)	2.32 (2)	3.0504 (13)	144.4 (16)
N1—H1⋯O2^i^	0.901 (17)	2.117 (17)	2.8978 (13)	144.4 (15)
N1—H1⋯O3^i^	0.901 (17)	2.303 (18)	3.0555 (13)	140.9 (15)
N2—H3⋯O3	0.818 (19)	2.161 (19)	2.9337 (14)	157.7 (17)
N3—H6⋯O2^i^	0.79 (2)	2.21 (2)	2.9016 (14)	146.3 (18)
N3—H5⋯O2^ii^	0.88 (2)	2.14 (2)	2.9586 (13)	154.4 (17)

Symmetry codes: (i) 
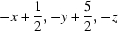
; (ii) 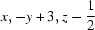.

**Fig. 1.**
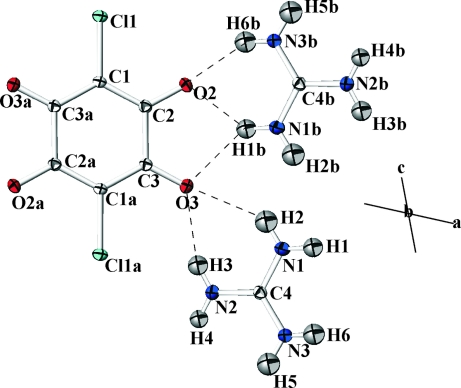
Molecular structure and atom naming scheme. Displacement ellipsoids are drawn at the 50% probability level. Grown fragment generated by symmetry codes: (a) - x, 2 - y, - z; (b) 1/2 - x, 2.5 - y, - z.

**Fig. 2.**
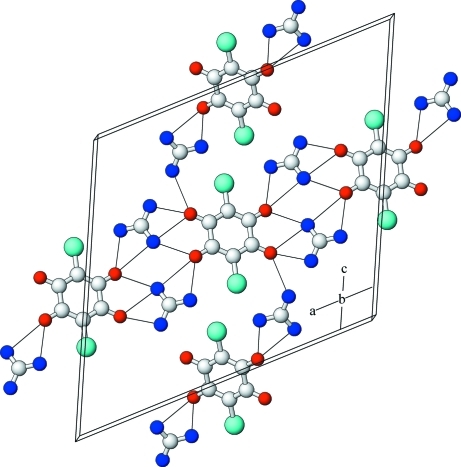
Packing diagram of the hydrogen-bonded framework structure of co-crystals viewed down the b axis, showing hydrogen-bonding associations as thin lines (H atoms are omitted).

## supplementary crystallographic information

### Comment

The co-crystallization of chloranilic acid and guanidine was carried out in methanol resulting in the co-crystal 2CH_6_N_3_^+^.C_6_O_4_Cl_2_^2-^ (Fig. 1). The chloranilic acid molecule is centro-symmetric and contains two hydrogen bond donors and two hydrogen bond acceptors and is, therefore, capable of participating in multiple hydrogen bonds. It forms 2D-sheet-like networks through N3—H5···O2 hydrogen bonds as shown in Figure 2. There are three N—H bonds in guanidine that connect with the O2 and O3 atoms of the chloranilic acid and form a one-dimensional molecular supramolecular structure. Two of these one-dimensional structures are again connected *via* N3—H5···O2 hydrogen bonds and form a two-dimensional network. Details of hydrogen-bonds are shown in Table 1.

### Experimental

Crystals were grown by slow evaporation of a methanol solution under ambient conditions containing a 1:1 stoichiometric quantity of guanidinium carbonate (Aldrich, 98%) and chloranilic acid (Aldrich, 99%).

### Refinement

Hydrogen atoms were found from the difference electron density maps and refined with isotropic temperature factors.

### Crystal data

**Table:**

2CH_6_N_3_^+^·C_6_Cl_2_O_4_^2^^−^	*F*(000) = 672
*M**_r_* = 327.14	*D*_x_ = 1.804 Mg m^−^^3^
Monoclinic, *C*2/*c*	Mo *K*α radiation, λ = 0.71070 Å
Hall symbol: -C 2yc	Cell parameters from 220 reflections
*a* = 19.5224 (14) Å	θ = 4.0–29.0°
*b* = 3.7316 (3) Å	µ = 0.57 mm^−^^1^
*c* = 18.4103 (14) Å	*T* = 173 K
β = 116.087 (1)°	Block, yellow
*V* = 1204.56 (16) Å^3^	0.45 × 0.40 × 0.30 mm
*Z* = 4	

### Data collection

**Table:**

Bruker SMART 1000 CCD diffractometer	1674 independent reflections
Radiation source: fine-focus sealed tube	1525 reflections with *I* > 2σ(*I*)
graphite	*R*_int_ = 0.020
ω scans	θ_max_ = 29.6°, θ_min_ = 2.3°
Absorption correction: multi-scan (*SADABS*, Sheldrick, 1996)	*h* = −27→27
*T*_min_ = 0.785, *T*_max_ = 0.849	*k* = −5→5
6965 measured reflections	*l* = −25→25

### Refinement

**Table:**

Refinement on *F*^2^	Primary atom site location: structure-invariant direct methods
Least-squares matrix: full	Secondary atom site location: difference Fourier map
*R*[*F*^2^ > 2σ(*F*^2^)] = 0.026	Hydrogen site location: inferred from neighbouring sites
*wR*(*F*^2^) = 0.078	All H-atom parameters refined
*S* = 1.08	*w* = 1/[σ^2^(*F*_o_^2^) + (0.0494*P*)^2^ + 0.7637*P*] where *P* = (*F*_o_^2^ + 2*F*_c_^2^)/3
1674 reflections	(Δ/σ)_max_ < 0.001
116 parameters	Δρ_max_ = 0.47 e Å^−^^3^
0 restraints	Δρ_min_ = −0.25 e Å^−^^3^

### Special details

**Table:**

Geometry. All e.s.d.'s (except the e.s.d. in the dihedral angle between two l.s. planes) are estimated using the full covariance matrix. The cell e.s.d.'s are taken into account individually in the estimation of e.s.d.'s in distances, angles and torsion angles; correlations between e.s.d.'s in cell parameters are only used when they are defined by crystal symmetry. An approximate (isotropic) treatment of cell e.s.d.'s is used for estimating e.s.d.'s involving l.s. planes.
Refinement. Refinement of *F*^2^ against ALL reflections. The weighted *R*-factor *wR* and goodness of fit *S* are based on *F*^2^, conventional *R*-factors *R* are based on *F*, with *F* set to zero for negative *F*^2^. The threshold expression of *F*^2^ > σ(*F*^2^) is used only for calculating *R*-factors(gt) *etc*. and is not relevant to the choice of reflections for refinement. *R*-factors based on *F*^2^ are statistically about twice as large as those based on *F*, and *R*- factors based on ALL data will be even larger.

### Fractional atomic coordinates and isotropic or equivalent isotropic displacement parameters (Å^2^)

**Table:**

	*x*	*y*	*z*	*U*_iso_*/*U*_eq_	
Cl1	0.036370 (14)	0.71393 (8)	0.174949 (15)	0.01677 (10)	
O2	0.14122 (4)	1.0852 (3)	0.12281 (5)	0.01823 (18)	
O3	0.11119 (5)	1.3347 (3)	−0.02492 (5)	0.01949 (19)	
N1	0.23429 (6)	1.5093 (3)	−0.07912 (6)	0.0213 (2)	
H2	0.2183 (11)	1.461 (6)	−0.0438 (12)	0.037 (5)*	
H1	0.2810 (10)	1.436 (5)	−0.0711 (10)	0.028 (4)*	
N2	0.11581 (6)	1.7324 (3)	−0.16016 (7)	0.0201 (2)	
H4	0.0871 (10)	1.852 (5)	−0.1996 (11)	0.024 (4)*	
H3	0.1026 (10)	1.648 (5)	−0.1273 (11)	0.030 (5)*	
N3	0.21154 (6)	1.7664 (3)	−0.20145 (7)	0.0211 (2)	
H6	0.2549 (12)	1.736 (5)	−0.1912 (12)	0.034 (5)*	
H5	0.1782 (11)	1.824 (6)	−0.2505 (13)	0.039 (5)*	
C1	0.01581 (6)	0.8726 (3)	0.07862 (6)	0.0143 (2)	
C2	0.07504 (6)	1.0395 (3)	0.06811 (6)	0.0140 (2)	
C3	0.05815 (6)	1.1811 (3)	−0.01681 (6)	0.0139 (2)	
C4	0.18720 (6)	1.6695 (3)	−0.14746 (7)	0.0154 (2)	

### Atomic displacement parameters (Å^2^)

**Table:**

	*U*^11^	*U*^22^	*U*^33^	*U*^12^	*U*^13^	*U*^23^
Cl1	0.01745 (15)	0.02061 (16)	0.01101 (15)	−0.00081 (9)	0.00514 (11)	0.00188 (9)
O2	0.0120 (3)	0.0267 (4)	0.0132 (4)	−0.0009 (3)	0.0030 (3)	0.0000 (3)
O3	0.0147 (4)	0.0274 (5)	0.0165 (4)	−0.0040 (3)	0.0069 (3)	0.0016 (3)
N1	0.0181 (5)	0.0293 (5)	0.0165 (5)	0.0044 (4)	0.0077 (4)	0.0056 (4)
N2	0.0150 (4)	0.0270 (5)	0.0191 (5)	0.0023 (4)	0.0082 (4)	0.0045 (4)
N3	0.0154 (5)	0.0326 (6)	0.0154 (5)	0.0012 (4)	0.0069 (4)	0.0049 (4)
C1	0.0139 (4)	0.0182 (5)	0.0099 (4)	−0.0003 (4)	0.0043 (4)	0.0014 (4)
C2	0.0133 (4)	0.0161 (5)	0.0119 (4)	0.0007 (4)	0.0051 (4)	−0.0008 (4)
C3	0.0129 (5)	0.0166 (5)	0.0120 (5)	0.0004 (4)	0.0053 (4)	0.0000 (4)
C4	0.0142 (5)	0.0168 (5)	0.0142 (5)	−0.0013 (4)	0.0052 (4)	−0.0015 (4)

### Geometric parameters (Å, °)

**Table:**

Cl1—C1	1.7409 (11)	N2—H3	0.818 (19)
O2—C2	1.2522 (13)	N3—C4	1.3267 (15)
O3—C3	1.2487 (13)	N3—H6	0.79 (2)
N1—C4	1.3297 (15)	N3—H5	0.88 (2)
N1—H2	0.85 (2)	C1—C2	1.3997 (14)
N1—H1	0.901 (17)	C1—C3^i^	1.4051 (14)
N2—C4	1.3287 (14)	C2—C3	1.5423 (15)
N2—H4	0.827 (18)	C3—C1^i^	1.4052 (14)
			
C4—N1—H2	118.9 (13)	C3^i^—C1—Cl1	118.10 (8)
C4—N1—H1	121.3 (11)	O2—C2—C1	124.91 (10)
H2—N1—H1	119.6 (17)	O2—C2—C3	116.98 (9)
C4—N2—H4	120.2 (12)	C1—C2—C3	118.10 (9)
C4—N2—H3	116.7 (13)	O3—C3—C1^i^	125.41 (10)
H4—N2—H3	123.1 (17)	O3—C3—C2	117.36 (9)
C4—N3—H6	118.7 (14)	C1^i^—C3—C2	117.23 (9)
C4—N3—H5	119.3 (13)	N3—C4—N2	120.90 (11)
H6—N3—H5	121.0 (18)	N3—C4—N1	120.33 (11)
C2—C1—C3^i^	124.66 (10)	N2—C4—N1	118.76 (11)
C2—C1—Cl1	117.24 (8)		

Symmetry codes: (i) −*x*, −*y*+2, −*z*.

### Hydrogen-bond geometry (Å, °)

**Table:**

*D*—H···*A*	*D*—H	H···*A*	*D*···*A*	*D*—H···*A*
N1—H2···O3	0.85 (2)	2.32 (2)	3.0504 (13)	144.4 (16)
N1—H1···O2^ii^	0.901 (17)	2.117 (17)	2.8978 (13)	144.4 (15)
N1—H1···O3^ii^	0.901 (17)	2.303 (18)	3.0555 (13)	140.9 (15)
N2—H3···O3	0.818 (19)	2.161 (19)	2.9337 (14)	157.7 (17)
N3—H6···O2^ii^	0.79 (2)	2.21 (2)	2.9016 (14)	146.3 (18)
N3—H5···O2^iii^	0.88 (2)	2.14 (2)	2.9586 (13)	154.4 (17)

Symmetry codes: (ii) −*x*+1/2, −*y*+5/2, −*z*; (iii) *x*, −*y*+3, *z*−1/2.
